# In Situ Coupling Carbon Defective C_3_N_5_ Nanosheet with Ag_2_CO_3_ for Effective Degradation of Methylene Blue and Tetracycline Hydrochloride

**DOI:** 10.3390/nano12152701

**Published:** 2022-08-05

**Authors:** Guoyu Li, Genying Zeng, Zhangkai Chen, Jiaming Hong, Xiaodong Ji, Zhiqiang Lan, Xiaofei Tan, Meifang Li, Xinjiang Hu, Chunfang Tang

**Affiliations:** 1 College of Environmental Science and Engineering, Central South University of Forestry and Technology, Changsha 410004, China; 2 College of Environmental Science and Engineering, Hunan University, Changsha 410082, China; 3 Key Laboratory of Environmental Biology and Pollution Control, Hunan University, Ministry of Education, Changsha 410082, China

**Keywords:** n-C_3_N_5_/Ag_2_CO_3_, photocatalysts, Z-scheme heterojunction, methylene blue, tetracycline hydrochloride, water remediation

## Abstract

The development of novel catalysts for degrading organic contaminants in water is a current hot topic in photocatalysis research for environmental protection. In this study, C_3_N_5_ nanosheet/Ag_2_CO_3_ nanocomposites (CNAC-X) were used as efficient photocatalysts for the visible-light-driven degradation of methylene blue (MB), and tetracycline hydrochloride (TC-HCl) was synthesized for the first time using a simple thermal oxidative exfoliation and in situ deposition method. Due to the synergistic effect of nanosheet structures, carbon defects, and Z-scheme heterojunctions, CNAC-10 exhibited the highest photocatalytic activity, with photodegradation efficiencies of 96.5% and 97.6% for MB (60 mg/L) and TC-HCl (50 mg/L) within 90 and 100 min, respectively. The radical trapping experiments showed that ·O_2_^−^ and h^+^ played major roles in the photocatalytic effect of the CNAC-10 system. Furthermore, intermediates in the photodegradation of MB and TC-HCl were investigated to determine possible mineralization pathways. The results indicated that C_3_N_5_ nanosheet/Ag_2_CO_3_ photocatalysts prepared in this work could provide an effective reference for the treatment of organic wastewater.

## 1. Introduction

Water pollution is a major worldwide environmental issue, and organic pollutants such as dyes and antibiotics possess a significant impact on the environment and human health [1,2]. According to previous reports, approximately 700,000 tons of various synthetic dyes are released into the water environment each year [3]. Methylene blue is one of the most common industrial azo dyes. These organic dyes and their intermediates usually have complex and stable aromatic structures, making them difficult to be degraded naturally. They are also carcinogenic and can cause a variety of dysfunctions in humans [4,5,6]. Tetracycline is a typical broad-spectrum antibiotic and is now widely used in the control of bacterial infections as well as livestock breeding and agricultural production activities [1,7,8,9,10]. However, due to the characteristics of antibiotics, they are not fully utilized in humans and animals, and a large amount of unmetabolized antibiotic residues released has a negative impact on the ecosystem and biological health [8,9,11,12]. In order to effectively eliminate organic pollutants from water, many researchers have explored a wide range of wastewater treatment technologies, such as physical adsorption [13], microbial degradation [14], electrochemical methods [15], and photocatalytic degradation [16,17]. However, physical adsorption only transfers organic pollutants from the aqueous phase to other phases without mineralizing them [18]; microbial degradation is limited by the high chemical stability of organic matter and its strong bactericidal ability [14]; and electrochemical methods can effectively mineralize organic matter but require continuous power input, leading to increased energy consumption and costs [15].

Currently, photocatalytic degradation based on semiconductor materials has become a promising green technology for organic removal due to its effective decomposition of organic pollutant molecules. Compared with traditional wastewater treatment technology, photocatalysis has the advantages of stronger degradation ability, higher mineralization rate, no secondary pollution, more simple operation, and lower cost [19]. In photocatalytic systems, semiconductor photocatalysts generate electron-hole pairs via electron leap under light excitation and further produce reactive oxygen species (ROS) such as ·OH and ·O_2_^−^ through a series of redox reactions, resulting in pollutant degradation and mineralization [20]. Therefore, the efficiency of photocatalytic reactions to degrade pollutants is largely determined by the performance of semiconductor catalysts containing energy band structure and surface structure, and it is critical to select a semiconductor material with superior photocatalytic activity [21].

Carbon nitride (CN) materials, due to their unique properties, such as low cost, metal-free, good visible light response, as well as controllable band and surface structure, have received significant attention [22]. Compared to g-C_3_N_4_ (*Eg* = 2.7 eV), widely reported previously, g-C_3_N_5_, as a novel carbon nitride material, exhibits superior photocatalytic activity due to more nitrogen-rich groups being introduced into triazole units. These nitrogen-rich groups change the C/N ratio in the molecular structure and expand the original π–π conjugation framework, resulting in a narrower optical band gap and more abundant electron sites in g-C_3_N_5_ [23,24,25,26,27]. However, similarly to other single semiconductors used in photocatalysis, the application of unmodified g-C_3_N_5_ is still constrained by factors such as the high complexation rate of photogenerated carriers and small specific surface area. To address these shortcomings, many researchers have reported various g-C_3_N_5_ modification methods, such as surface structure modification [22,28], modulation of energy band structure [29], elemental doping [25], and heterojunction construction [30,31]. Among these modification strategies, coupling with other semiconductors to form heterojunction can effectively improve the performance of photocatalysts [32]. In recent years, a large number of silver-based nanomaterials, such as AgCl [33], Ag_2_CrO_4_ [34], Ag_3_PO_4_ [35], and AgVO_3_ [36], have been shown to be novel photocatalysts with visible light responsiveness. Ag_2_CO_3_ is a p-type semiconductor photocatalyst with a narrow band gap and good visible light activity, and it has gained extensive attention for its excellent degradation performance against organic pollutants [37]. However, silver-based nanomaterials are susceptible to photocorrosion when exposed to visible light, which limits their catalytic performance and stability. The CN material can be coupled with silver-based nanomaterial to form a heterojunction structure, which not only improves the separation rate of photogenerated carriers and further enhances the photocatalytic activity, but also effectively suppresses the photocorrosion of silver-based nanomaterials [38]. Furthermore, the introduction of structural defects has been proven to induce the capture of photogenerated electrons and reduce the complexation with holes, thus ensuring the effective separation of photogenerated carriers [32].

To the best of our knowledge, there is no report on coupling C_3_N_5_ nanosheets with Ag_2_CO_3_. Therefore, we report a nitrogen-rich C_3_N_5_ nanosheet/Ag_2_CO_3_ Z-scheme heterojunction with carbon defects prepared by thermal oxidative exfoliation and in situ deposition. The microstructure, crystalline phase, surface chemistry, optical properties, and electron transfer pathways of the catalysts were investigated by characterization and a series of photodegradation experiments on MB and TC-HCl. Furthermore, radical trapping experiments and intermediate product analysis were used to elucidate the degradation mechanisms of MB and TC-HCl. In conclusion, the novel photocatalyst developed in this work can generate ROS to treat organic wastewater by depending only on visible light as the energy source. Therefore, this study is in accordance with the current concept of combining energy conservation and environmental management and can provide an effective reference for water pollution control.

## 2. Experiment

### 2.1. Materials

3-amino-1,2,4-triazole (3-AT), melamine, isopropanol (IPA), p-benzoquinone (BQ), triethanolamine (TEOA), and tetracycline hydrochloride (TC-HCl) were purchased from Macklin (Shanghai, China). AgNO_3_, NaHCO_3_, ethanol, and methylene blue (MB) were produced by Sinopharm chemical reagent Co., Ltd. (Shanghai, China). All reagents used in this experiment were of analytical grade without further refinement. Ultrapure water (18.25 mΩ/cm) was used throughout the study.

### 2.2. Synthesis of g-C_3_N_4_

Typically, 5 g of melamine was put into an Al_2_O_3_ crucible with a cover and then heated to 500 °C for 3 h at a heating rate of 3 °C/min in a muffle oven. The resulting yellow sample was ground into powder for the subsequent experiment.

### 2.3. Synthesis of g-C_3_N_5_

The synthesis method of g-C_3_N_5_ was based on a previous study [39]. Briefly, 5 g of 3-AT was added into an Al_2_O_3_ crucible with a half-cover state and then heated to 500 °C for 3 h at a heating rate of 3 °C/min in a muffle oven. The resulting brown sample was ground into powder for the subsequent experiment.

### 2.4. Synthesis of g-C_3_N_5_ Nanosheet (n-C_3_N_5_)

g-C_3_N_5_ with a nanosheet structure (n-C_3_N_5_) was prepared by thermal exploitation of the g-C_3_N_5_ obtained above. Typically, 1 g of g-C_3_N_5_ was uniformly dispersed into an open crucible to keep g-C_3_N_5_ in full contact with air. The crucible was heated in a muffle furnace at 500 °C for 1 h followed by 520 °C for 2 h at a heating rate of 3 °C/min. The product was cooled naturally in the muffle furnace, and the obtained pale-yellow powder was n-C_3_N_5_.

### 2.5. Synthesis of n-C_3_N_5_/Ag_2_CO_3_ Composite (CNAC)

**Figure 1:**
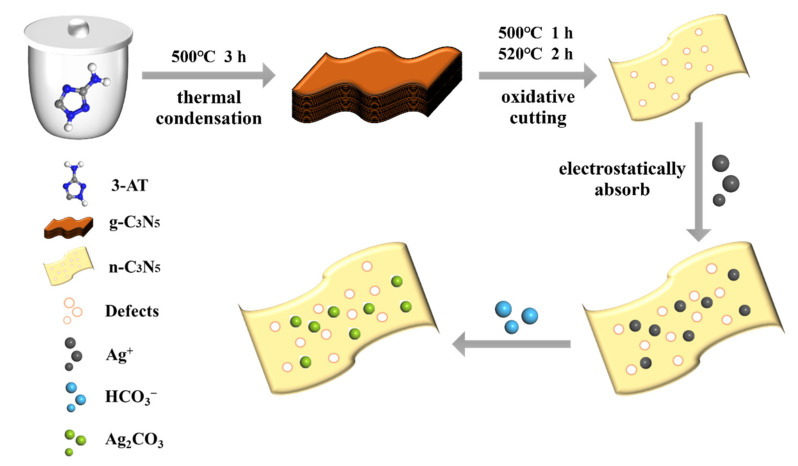
Illustration of the fabrication procedure of the n-C_3_N_5_/Ag_2_CO_3_ (CNAC) composite.

The n-C_3_N_5_/Ag_2_CO_3_ composites with different mass ratios were produced via an in situ chemical precipitation reaction (Figure 1). Firstly, 0.3 g of as-prepared n-C_3_N_5_ was dispersed in 100 mL of ultrapure water and continuously sonicated for 3 h to form a suspension. Secondly, a certain amount of AgNO_3_ solution (0.037 g, 10 mL) was added to the above suspension and stirred for 1 h under dark conditions to ensure the adsorption equilibrium of Ag^+^ on the surface of n-C_3_N_5_. Thirdly, a NaHCO_3_ aqueous solution (0.018 g, 20 mL) was added dropwise to the above mixture of Ag^+^/n-C_3_N_5_ at a rate of 10 mL/h, and the mixture was stirred continuously for 18 h in the dark to facilitate the growth of Ag_2_CO_3_ nanoparticles. Lastly, the resulting precipitate was collected by centrifugation, washed three times with ultrapure water and ethanol, and dried at 60 °C in a vacuum oven for 12 h. The as-prepared complex was denoted as CNAC-10. For comparison, the CNAC-X (X represents the weight percentages of Ag_2_CO_3_ in CNAC) with different mass ratios were prepared by controlling the amount of AgNO_3_ and NaHCO_3_, referred to as CNAC-5, CNAC-10, CNAC-15, and CNAC-20. The bare Ag_2_CO_3_ was also fabricated without the addition of n-C_3_N_5_.

### 2.6. Characterization

The morphology of the as-prepared samples was observed by field emission scanning electron microscopy (SEM; Sigma 300, ZEISS, Oberkochen, Germany) with an acceleration voltage of 3 kV. The specific surface areas were investigated using the Brunauer–Emmett–Teller (BET) method using a nitrogen absorption analyzer (Autosorb IQ3, Quantachrome, Boynton Beach, FL, USA). The crystal pattern information of samples was investigated by X-ray diffraction (XRD; Smartlab SE, Rigaku, Japan) with Cu Kα radiation at 40 kV and 40 mA (λ = 1.54 Å) at a scanning speed of 2 °/min. Fourier transform infrared (FT-IR) spectroscopy (400–4000 cm^−1^) was performed using Vertex 70 (Bruker, Bremen, Germany). Electron paramagnetic resonance (EPR) was measured at room temperature on an EMXplus-6/1 wave spectrometer (Bruker, Bremen, Germany). Electron spin resonance (ESR) signals were detected with a JES FA200 spectrometer (JEOL, Tokyo, Japan). X-ray photoelectron spectroscopy (XPS; Thermo ESCALAB 250XI, Waltham, MA, USA; Measuring voltage 16 kV; Current 14.9 mA; Beam spot 650 μm; Charge calibration with C 1s = 284.8 eV) was used to analyze the chemical states of photocatalysts. The Ultraviolet-visible diffuse reflectance spectra (UV-vis DRS) were probed on a Lambda 750S spectrophotometer (PerkinElmer, Waltham, MA, USA). Photoluminescence (PL, FLS1000, Edinburgh, UK) spectra were measured with an excitation wavelength of 340 nm. The photodegradation pathways of MB and TC-HCl were analyzed by Ultimate 3000 UHPLC-Q Exactive high-performance liquid chromatography-tandem mass spectrometry (HPLC-MS, Thermo Scientific, Waltham, MA, USA) with a C18 column (250 mm × 4.6 mm, 5 μm). Mobile phase A was 0.1% formic acid; mobile phase B was acetonitrile. The gradient A:B = 95:5, the flow rate was 0.8 mL/min, and the column temperature was 30 °C. The Mass Spectrometry Analyzer was Thermo Scientific Q Exactive (Warping rate = 40 mL/min; Auxiliary gas rate = 10 mL/min; positive ion 4 kV; Capillary temperature = 300 °C).

### 2.7. Photoelectrochemical Measurements

The transient photocurrent responses (I-t curves) and electrochemical impedance spectroscopy (EIS) were performed on a CHI 760E workstation with a three-electrode system. In the electrode model, the counter electrode is the Pt electrode, the reference electrode is the Ag/AgCl electrode, and the working electrode is ITO conductive glass. The test procedure of photoelectrochemistry is described as follows. In total, 10 mg of the catalyst was dispersed in 1 mL of 0.25% Nafion solution and sonicated for 30 min to form a homogeneous suspension. Then, the 150 μL suspension was added dropwise on ITO glass and dried at room temperature for photoelectricity testing.

### 2.8. Photocatalytic Experiments

The photocatalytic performance of the resulting photocatalysts was evaluated via simulated visible light irradiation (λ > 400 nm) by a 300 W xenon lamp using methylene blue (MB) and tetracycline hydrochloride (TC-HCl) as target contaminants. Firstly, 50 mg of catalyst was added into MB (60 mg/L, 50 mL, and pH = 8.0) solution, and 20 mg of catalyst was added into TC-HCl (50 mg/L, 50 mL, and pH = 4.8) solution. Then, the two mixtures were sonicated for 5 min to ensure the catalyst was uniformly dispersed. The solutions were magnetically stirred under dark conditions for 30 min to reach adsorption equilibrium before visible light treatment. During illumination, 2 mL of the suspension was taken out at intervals and passed through a 0.22 μm filter to remove the photocatalyst. The residual concentrations of MB and TC-HCl in the filtrate were determined using an ultraviolet-visible spectrophotometer (UV-2700, Shimadzu, Japan) at 664 and 357 nm, respectively.

## 3. Results and Discussion

### 3.1. Phase and Microstructure of Photocatalysts

#### 3.1.1. SEM, EDX and BET Analysis

**Figure 2:**
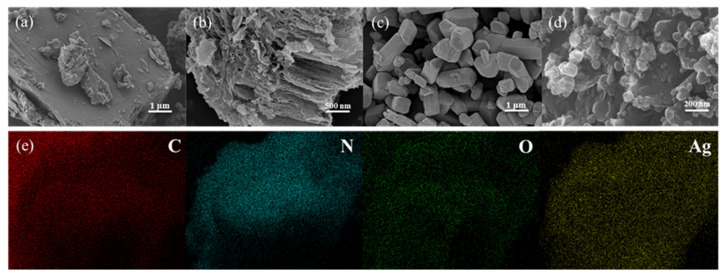
SEM images of (**a**) g-C_3_N_5_, (**b**) n-C_3_N_5_, (**c**) Ag_2_CO_3_, and (**d**) CNAC-10; (**e**) EDX mappings of C, N, O, and Ag elements for CNAC-10.

The structures and microscopic morphologies of bulk g-C_3_N_5_, n-C_3_N_5_, Ag_2_CO_3_, and CNAC-10 were analyzed by field emission scanning electron microscopy (FE-SEM) (Figure 2a–d). The morphology of pristine g-C_3_N_5_ is relatively smooth with a tightly stacked rock-like structure, whereas n-C_3_N_5_ has a fluffy flake-like structure with a large number of mesopores and a significant reduction in planar size, indicating a tight overall structure of C_3_N_5_ was successfully stripped. The nanosheet-like and mesoporous structures not only increased the specific surface area and active sites of the catalysts but also improved the visible light absorption and accelerated the transfer of photogenerated charges, thereby boosting photocatalytic activity [40]. The BET analysis further confirmed that thermal exfoliation is an effective way to fabricate C_3_N_5_ nanosheets with a large specific surface area. The N_2_ adsorption–desorption curve (Figure S1) shows that the specific surface area of n-C_3_N_5_ increased remarkably from 10.6 m^2^/g to 177.9 m^2^/g compared with that of g-C_3_N_5_. As for pure Ag_2_CO_3_, it can be observed that it is composed of numerous polyhedral rod-like particles with an average size of 0.2–1 μm and a smooth surface. In terms of CNAC-10, the Ag_2_CO_3_ nanoparticles are tightly attached to the surface of n-C_3_N_5_, forming a heterogeneous junction structure. Meanwhile, the size of Ag_2_CO_3_ nanoparticles slightly decreased, which may be due to the improving dispersion of Ag_2_CO_3_ in the nanosheet structure of n-C_3_N_5_ [30]. The EDX pattern of CNAC-10 (Figure 2e) clearly reveals the distribution of C, N, O, and Ag elements in the CNAC-10 composite, confirming the formation of binary materials.

#### 3.1.2. XRD Analysis

**Figure 3:**
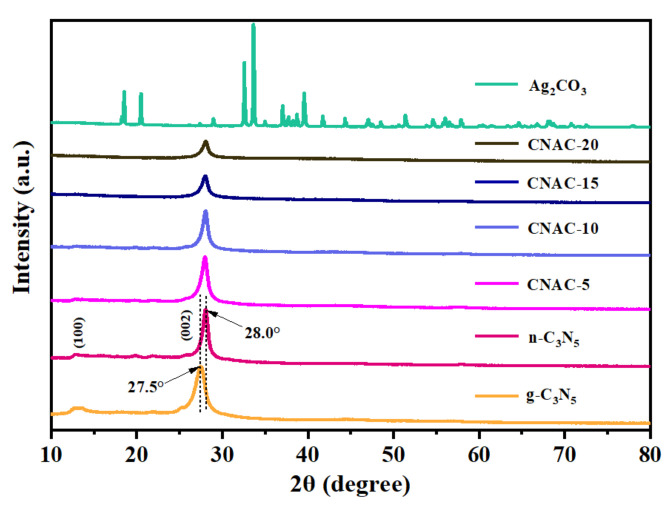
XRD patterns of as-prepared photocatalysts.

X-ray diffraction (XRD) was used to analyze the crystal pattern information of the synthesized catalysts (Figure 3). The bulk g-C_3_N_5_ presented two distinct diffraction peaks at 2θ = 13.5° and 27.5°, corresponding to the (100) and (002) planes of the carbon nitride material, respectively [41]. The weak peak at 13.5° referred to an in-plane repeating triazole ring in the g-C_3_N_5_ structure, while the main peak at 27.5° was related to the tight interlayer stacking structure of the aromatic system; these results are consistent with previous reports [23]. Moreover, we discovered that the (002) peak of n-C_3_N_5_ shifts to 28.0°, corresponding to a reduction in the interlayer stacking distance in the n-C_3_N_5_ structure, which facilitates the acceleration of photogenerated charge transfer [39]. The decrease of the diffraction peak at 13.5° also confirms the successful exfoliation of the n-C_3_N_5_ interlayer stacking structure, resulting in smaller planar size compared to g-C_3_N_5_ [28]. As for pure Ag_2_CO_3_, all the diffraction peaks correspond to the standard monoclinic phase of Ag_2_CO_3_ (JCPDS NO. 26-0339), with sharp diffraction peaks and no other impurity peaks detected, indicating the good crystallinity and high purity of the prepared Ag_2_CO_3_ [42]. However, no Ag_2_CO_3_ diffraction peaks were found in CNAC-X composites, probably due to the low content or high dispersity of Ag_2_CO_3_ particles grown in situ on the surface of n-C_3_N_5_ nanosheets, resulting in weak diffraction intensity [43,44].

#### 3.1.3. FTIR and EPR Analysis

**Figure 4:**
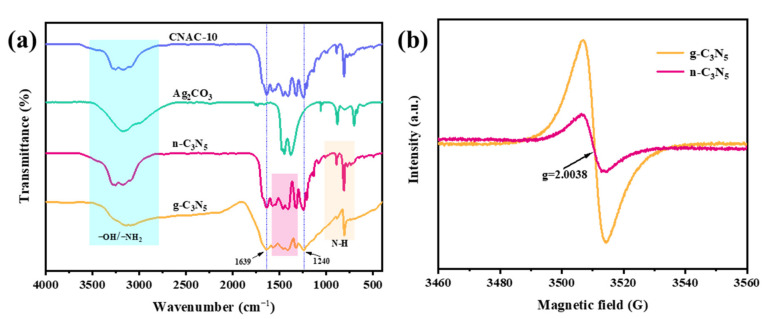
(**a**) The FTIR spectra of g-C_3_N_5_, n-C_3_N_5_, Ag_2_CO_3_, and CNAC-10; (**b**) the EPR spectra of g-C_3_N_5_ and n-C_3_N_5_.

Fourier transform infrared (FT-IR) spectroscopy was used to investigate the surface functional groups and molecular structures of the samples (Figure 4a). For g-C_3_N_5_, broad peaks in the range of 2750 to 3500 cm^−1^ are attributed to residual -NH_2_ groups on the catalyst surface or -OH bond stretching vibration of adsorbed water molecules [25]. The peaks at 1639 and 1240 cm^−1^ are associated with the stretching vibrations of C=N and C-N bonds, respectively [23]. The absorption peaks originating from 1300 to 1560 cm^−1^ are related to the respiratory vibration of the triazine ring [39]. Additionally, the N-H bending vibration is responsible for the peak observed between 700 and 1000 cm^−1^ [29]. Compared to g-C_3_N_5_, an enhanced peak at 3164 cm^−1^ of the n-C_3_N_5_ nanosheet can be observed, corresponding to the stretched vibrational absorption peak of the terminal -NH_2_ in the n-C_3_N_5_ structure. In addition, the transmittance of N-H-related bands at 805 and 880 cm^−1^ also significantly increased, indicating that more N-H or -NH_2_ groups are generated in the framework of n-C_3_N_5_, probably due to the erosion of oxygen. The high-temperature oxidative exfoliation process could break the triazine structure of n-C_3_N_5_ and form C-NH_2_ groups, producing structural defects for trapping electrons and thus improving the separation of photogenerated carriers [45]. The results of EPR spectra (Figure 4b) provide direct evidence for the existence of structural defects. Both g-C_3_N_5_ and n-C_3_N_5_ exhibit a dominant EPR signal with a g value of 2.0038, which is related to the unpaired electrons of carbon atoms on the triazine ring [46]. Compared to g-C_3_N_5_, the EPR signal intensity of n-C_3_N_5_ is significantly lower, suggesting that carbon vacancies were introduced into the structure of n-C_3_N_5_, leading to a decrease in the number of unpaired electrons [45,47]. Regarding the spectrum of Ag_2_CO_3_, the absorption peaks located at 1448, 1376, 1058, 880, and 707 cm^−1^ are ascribed to CO_3_^2−^ stretching vibrations in carbonates [42,43,48]. However, the characteristic absorption peak of Ag_2_CO_3_ was not detected on the FT-IR spectra of the CNAC-10 composites, suggesting that the characteristic peak of low content or high dispersion of Ag_2_CO_3_ nanoparticles was covered up by the peak of n-C_3_N_5_. Additionally, a slight decrease in the diffraction peak intensity of n-C_3_N_5_ was observed, which can be explained by the chemical bonding interaction between n-C_3_N_5_ and Ag_2_CO_3_; therefore, the chemical environment of n-C_3_N_5_ was changed, which was also previously reported [44,49].

#### 3.1.4. XPS Analysis

**Figure 5:**
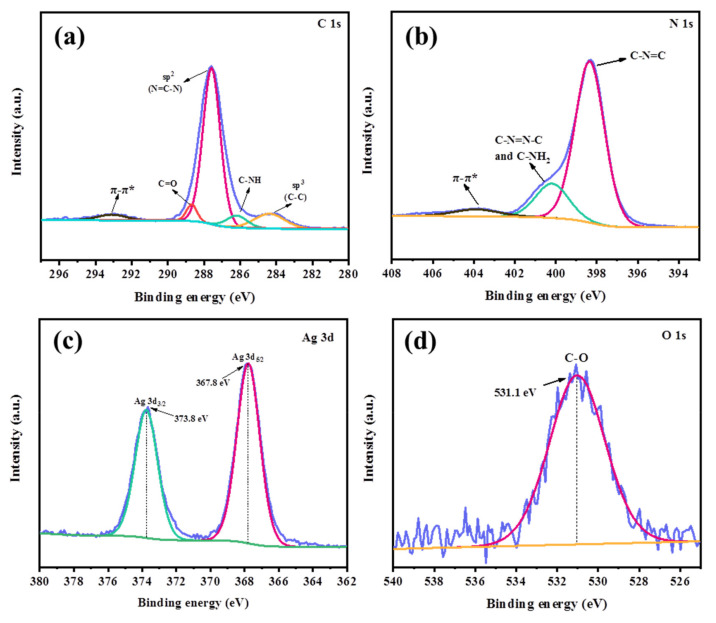
XPS spectra of CNAC-10, (**a**) C 1s, (**b**) N 1s, (**c**) Ag 3d, and (**d**) O 1s.

The surface elemental composition and chemical conformation of CNAC-10 samples were explored using XPS analysis. Figure S2 show the XPS measurement spectra of the CNAC-10 composites, where the dominant peaks of four elements, C, N, Ag, and O, can be clearly observed. In terms of the high-resolution XPS analysis of C 1s (Figure 5a), the peaks at 284.4, 286.2, 287.6, 288.6, and 293.1 eV are assigned to the sp^3^ hybridized carbon of the indeterminate carbon skeleton in n-C_3_N_5_, the C-NH group in the triazine structure, the aromatic sp^2^ hybridized N=C–N group constituting CN structure groups, C=O bonds in Ag_2_CO_3_ nanoparticles, and π-electron delocalization domains in C-N heterocycles, respectively [23,25,31,50,51]. From Figure 5b, three peaks located at 398.4, 400.2, and 403.9 eV can be observed in the N 1s spectrum after deconvolution. The tertiary [N-(C)_3_] and secondary (-C-N=C) nitrogen in the aromatic structure are represented by the peak at 398.4 eV, the peak at 400.2 eV is attributed to an aliphatic C-N=N-C or residual C-NH_2_ group, and the weak peak at 403.9 eV is associated with π-π* bonding [23,26,30]. The Ag 3d spectrum with two peaks detected at 367.8 and 373.8 eV is ascribed to the Ag 3d_5/2_ and Ag 3d_3/2_ binding energies of Ag^+^, respectively (Figure 5d), indicating the presence of Ag^+^ in CNAC-10 [42,48,52,53]. Additionally, as for the deconvoluted O 1s high-resolution spectrum (Figure 5d), the center of the peak is located at about 531.1 eV, corresponding to the C-O bond in Ag_2_CO_3_ [54]. Overall, XPS analysis confirmed the elemental chemical state and surface properties of CNAC-10 composites, further proving the successful fabrication of CNAC-10 samples.

### 3.2. Optical Properties of Photocatalysts

#### 3.2.1. UV-vis DRS Analysis

**Figure 6:**
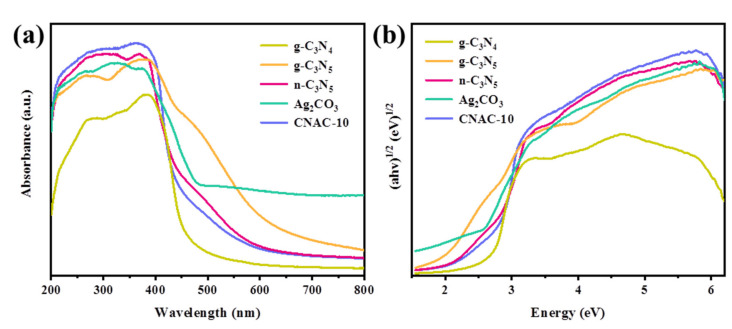
(**a**) UV–vis DRS spectra and (**b**) bandgap energy of photocatalysts.

The light absorption properties and energy band structures of g-C_3_N_4_, g-C_3_N_5_, n-C_3_N_5_, Ag_2_CO_3_, and CNAC-10 were determined by UV-vis diffuse reflectance spectroscopy (Figure 6a,b). The characteristic absorption peak of bare g-C_3_N_4_ is between 300–390 nm, with an absorption edge of about 460 nm, mainly for ultraviolet light [55]. Compared with g-C_3_N_4_, the absorption spectrum of g-C_3_N_5_ is red-shifted, and the band tail is extended to around 620 nm, indicating that g-C_3_N_5_ can absorb a larger range of visible light, and thus the photocatalytic activity is enhanced. The prolonged absorption edge of g-C_3_N_5_ is mainly owing to the overlap between N 2p orbitals in the aromatic system, which extends the π-conjugated structure [23,56]. Furthermore, the absorption band tail of n-C_3_N_5_ blue-shifted to approximately 570 nm with interlayer exfoliation. This blue-shifting trend was attributed to the quantum confinement effect of the semiconductor, implying the successful synthesis of n-C_3_N_5_ in a two-dimensional nanosheet structure by thermal oxidative exfoliation [43,45,57]. The absorption band edge of pure Ag_2_CO_3_, with strong absorption of visible light, is roughly 510 nm. After the Ag_2_CO_3_ nanoparticles were deposited on the surface of n-C_3_N_5_, the absorption band tail of the n-C_3_N_5_/Ag_2_CO_3_ heterostructure was blue-shifted, and the band gap was widened. Subsequently, the band gap values of g-C_3_N_4_, g-C_3_N_5_, n-C_3_N_5_, Ag_2_CO_3_, and CNAC-10 were calculated using the Tauc Equation (1) [23]:

(1) $${ahv=A(hv-Eg)}^{n/2}$$

where *a*, *h*, *v* represents the optical absorption coefficient, Planck’s constant, and optical frequency, while *A* and *Eg* refer to the defining constant and band gap value, respectively. The value of *n* is determined by the type of semiconductor leap (direct leap *n* = 1 and indirect leap *n* = 4). According to previous reports, g-C_3_N_4_, g-C_3_N_5_, and Ag_2_CO_3_ are indirect semiconductors, so their *n* values are 4 [39,54,55]. Therefore, the band gap values of g-C_3_N_4_, g-C_3_N_5_, n-C_3_N_5_, Ag_2_CO_3_, and CNAC-10 can be calculated as 2.70, 2.00, 2.18, 2.43, and 2.34 eV, respectively.

#### 3.2.2. PL Analysis

**Figure 7:**
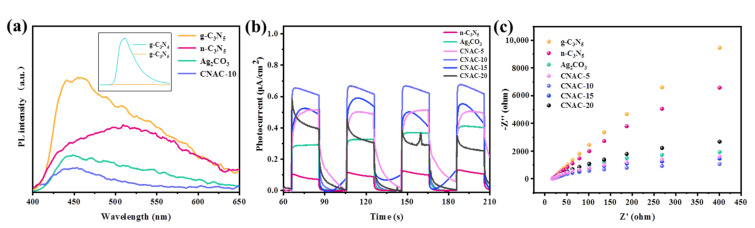
(**a**) PL spectra; (**b**) photocurrent response; and (**c**) EIS Nyquist plots.

The separation and migration efficiency of photogenerated charges have an important effect on the photocatalytic activity of photocatalysts. Photoluminescence (PL) spectroscopy was applied to explore the separation rate of photogenerated electron-hole pairs. Theoretically, photogenerated electrons tend to combine with holes under coulomb force to generate fluorescence. Therefore, a lower fluorescence emission intensity means a higher charge separation efficiency [58]. Previous reports showed that g-C_3_N_4_ had a high PL intensity [1]. Figure 7a show that the prepared g-C_3_N_5_ presents a lower emission intensity than g-C_3_N_4_, indicating a lower complexation rate of the photo-induced electron-hole pair on g-C_3_N_5_. In addition, due to the ultrathin structure and the presence of carbon defects after thermal oxidative stripping, the charge separation and transfer efficiency of n-C_3_N_5_ is significantly enhanced, and the PL intensity is markedly decreased. However, compared with monomeric materials, CNAC-10 composite exhibits the lowest PL intensity with the addition of Ag_2_CO_3_ nanoparticles, indicating the synergistic effects of nanosheet structure, carbon vacancies, and heterojunction structure all play important roles in enhancing the photogenerated charge separation rate and photocatalytic activity of photocatalysts.

#### 3.2.3. Photocurrent and EIS Analysis

To further test electron migration properties, the photocurrent response and electrochemical impedance of samples were measured on an electrochemical working device configured with visible light irradiation (λ > 400 nm). Figure 7b display the photocurrent curves of n-C_3_N_5_, Ag_2_CO_3_, CNAC-5, CNAC-10, CNAC-15, and CNAC-20, among which CNAC-10 exhibits the highest photocurrent intensity within four cycle periods. In general, a stronger photocurrent response corresponds to a faster electron migration rate [59]. This revealed that the CNAC-10 sample has a lower electron-hole pair complexation rate compared to n-C_3_N_5_, Ag_2_CO_3_, and other CNAC-X composites, thus possessing higher photocatalytic activity. Additionally, similar results are shown in the photoelectrochemical impedance spectra. As shown in Figure 7c, the CNAC-10 composite has a smaller Nyquist arc radius than g-C_3_N_5_, n-C_3_N_5_, Ag_2_CO_3_, and other CNAC-X composites. The charge transfer resistance of the catalyst is positively correlated with the arc radius on the electrochemical impedance spectrum [60,61]. Therefore, EIS analysis indicates that the CNAC-10 composite has lower resistance to photogenerated charge migration than other samples. In summary, the presence of the heterojunction structure and defects accelerated the interfacial charge transfer between n-C_3_N_5_ and Ag_2_CO_3_; thus, the photocatalytic activity of CNAC-10 was boosted.

### 3.3. Photocatalytic Activity

**Figure 8:**
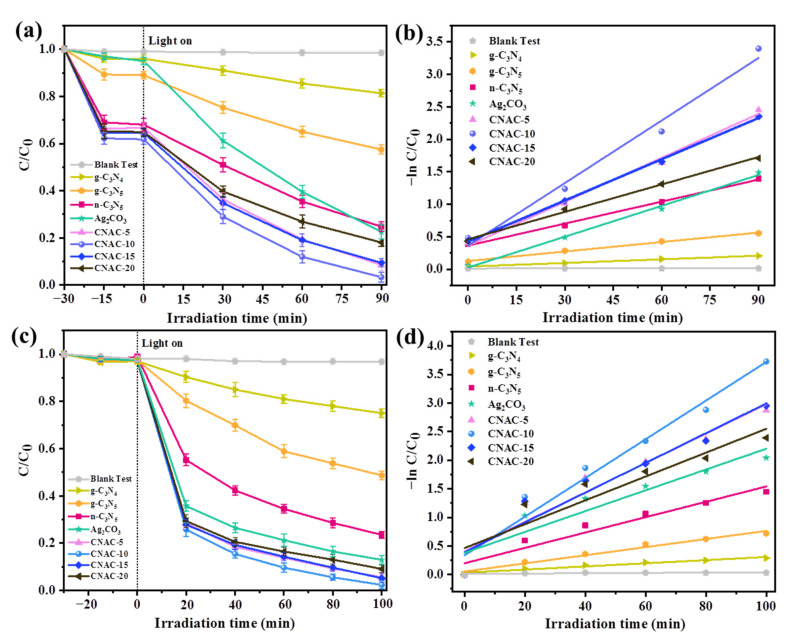
(**a**,**c**) The photodegradation of MB and TC-HCl in the presence of as-prepared catalysts under visible light irradiations and (**b**,**d**) corresponding reaction kinetic constants of different samples under visible light.

The degradation performance of prepared photocatalysts against MB and TC-HCl was investigated under visible light irradiation (λ > 400 nm) to evaluate their photocatalytic activity. Prior to photocatalytic degradation, the reaction solution containing photocatalyst and MB or TC-HCl was placed in dark conditions for the adsorption reaction for the desired time. It can be seen from Figure 8a,c that the adsorption phase of MB and TC-HCl on photocatalysts reached equilibrium at approximately 30 min. For MB, the adsorption efficiencies of g-C_3_N_4_, g-C_3_N_5_, n-C_3_N_5_, Ag_2_CO_3_, CNAC-5, CNAC-10, CNAC-15, and CNAC-20 were 4.0%, 11.4%, 32.3%, 5.3%, 33.3%, 38.2%, 35.6%, and 35.1%, respectively. However, the prepared photocatalysts had almost no adsorption effect on TC-HCl owing to electrostatic repulsion [23]. Subsequently, visible light degradation experiments were carried out by applying a 300 W xenon lamp, and the outcomes are also shown in Figure 8a,c. The blank experiment without the presence of photocatalyst proved that the concentrations of MB and TC-HCl were relatively unchanged, demonstrating that the chemical properties of MB and TC-HCl were stable and the direct photolysis of MB and TC-HCl by visible light irradiation could be neglected. Compared to g-C_3_N_4_, the prepared g-C_3_N_5_ exhibits superior photocatalytic performance, mainly attributed to its better visible light capture ability. Additionally, the degradation performance of n-C_3_N_5_ was further improved, with photodegradation rates of 75.2% and 76.4% for MB and TC-HCl, respectively, mainly benefiting from enhanced photogenerated charge separation efficiency by the ultrathin structure, and carbon defects generated by thermal oxidative stripping. In addition, the photocatalytic activity of CNAC-X composites was significantly enhanced with the introduction of Ag_2_CO_3_ nanoparticles. This is due to the formation of a heterojunction structure between n-C_3_N_5_ and Ag_2_CO_3_, which helps the charge transfer between both materials. Under visible light irradiation, the degradation rates of CNAC-5, CNAC-10, CNAC-15, and CNAC-20 were 91.4%, 96.5%, 90.5%, and 81.9% for MB in 90 min; while 94.3%, 97.6%, 94.7%, and 90.8% of TC-HCl were removed within 100 min. Therefore, the CNAC-10 sample exhibited the highest photodegradation efficiency for MB and TC-HCl. However, the photocatalytic performance of the composites decreased when the content of Ag_2_CO_3_ was further increased, probably because the active sites of the CNAC-X composites were occupied by the excess Ag_2_CO_3_ particles. Some representative photocatalyst composites for MB, TC-HCl or RhB degradation reported in recent years are summarized in Table S1 [23,30,31,43,62].

To further explore the photocatalytic activity of the samples, the kinetics of photocatalytic degradation of MB and TC-HCl were investigated using a pseudo-first-order model [Equation (2)]:

−ln (*C*/*C*_0_) = *k* × *t* (2)

where *C*_0_ and *C* represent the concentration of MB or TC-HCl at illumination times of 0 and *t*, and *k* is the reaction rate constant. Figure 8b,d illustrate the pseudo-level fitting curves for the removal processes of MB and TC-HCl by the synthesized photocatalysts. The degradation kinetic constant *k* of CNAC-10 for MB was 0.031 min^−1^, which was 2.82 and 1.94 times higher than that of n-C_3_N_5_ (0.011 min^−1^) and Ag_2_CO_3_ (0.016 min^−1^), respectively. The degradation kinetic constant *k* of CNAC-10 for TC-HCl is 2.54 and 1.89 times higher than that of n-C_3_N_5_ and Ag_2_CO_3_, respectively. In summary, the coordinated effect of nanosheet structure, carbon defects, and heterojunction structure plays a crucial role in the photocatalytic performance of CNAC-X.

**Figure 9:**
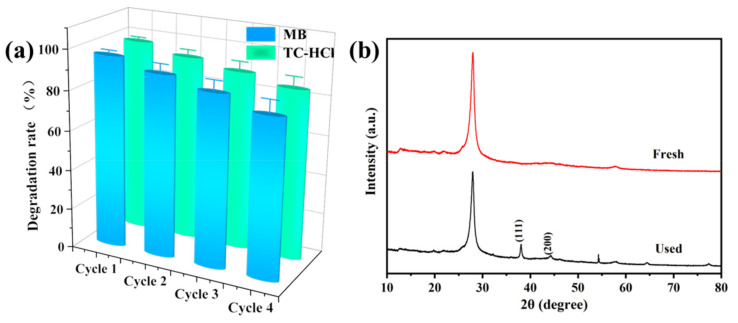
(**a**) Four recycling runs of the CNAC-10 for MB and TC-HCl degradation and (**b**) XRD patterns of fresh and used CNAC-10 after four recycling runs.

Moreover, cyclic photodegradation experiments of MB and TC-HCl were conducted to explore the recoverability and stability of CNAC-10. After each cycle, the photocatalysts were ultrasonically cleaned with ultrapure water and ethanol, recovered by high-speed centrifugation, and dried under vacuum at 60 °C for 4 h for subsequent recycling. As illustrated in Figure 9a, due to the inevitable catalyst loss, the removal rates of MB and TC-HCl by CNAC-10 decreased slightly after four consecutive reuses, from 96.5% to 80.1% and 97.6% to 85.4%, respectively. To further analyze the phase transition of the catalyst, the XRD diffractograms of CNAC-10 before and after four cycles of degradation were compared. Figure 9b demonstrate that the crystalline phase of the sample remains essentially unchanged. In addition, new characteristic peaks were detected at 38.0° and 44.2°, corresponding to the (111) and (200) crystal planes of metallic silver. This indicates that CNAC-10 was subjected to slight photocorrosion after four cycles, resulting in the partial photoreduction of Ag^+^ from Ag_2_CO_3_ nanoparticles to metallic silver [63]. In conclusion, the cycling experiments reveal that CNAC-10 has superior recyclability and stability and can be used as an efficient and durable photocatalyst for the photodegradation of MB and TC-HCl in water.

### 3.4. Photocatalytic Mechanism

#### 3.4.1. The Possible Degradation Pathways

**Figure 10:**
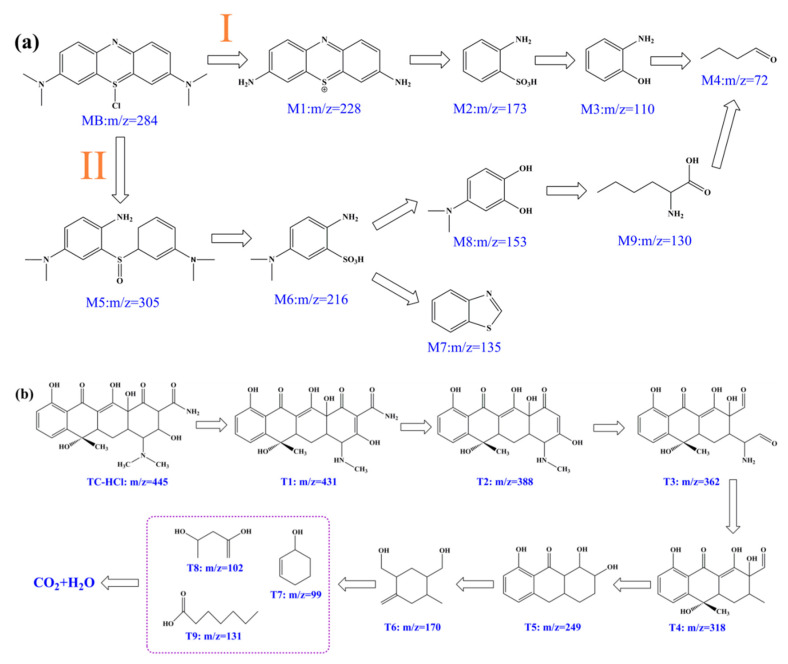
(**a**) The proposed photocatalytic degradation pathway of MB and (**b**) TC-HCl over CNAC-10.

In order to clarify the degradation pathways of MB and TC-HCl, the intermediates of their photocatalytic processes were determined by LC-MS. Figure S3a show the *m*/*z* values of MB photodegradation at 90 min in the presence of a CNAC-10 catalyst, and intermediate signal peaks with different *m*/*z* values can be observed. Based on the LC-MS data and previous reports, two possible degradation pathways of MB were proposed (Figure 10a) [64,65,66,67]. In pathway I, the original MB (*m*/*z* = 284) was converted to intermediate M1 (*m*/*z* = 228) by S-Cl bond breaking and demethylation processes, followed by deamination and desulfonation reactions to produce intermediates M2 (*m*/*z* = 173) and M3 (*m*/*z* = 110). Finally, it was transformed into a small molecule product M4 (*m*/*z* = 72) by an oxidative ring opening reaction. For pathway II, the MB molecule was converted to intermediate M5 (*m*/*z* = 305) via the breakage of S-Cl and N=C bonds and the formation of S=O bonds. Then, M5 underwent a sulfonation reaction and S-C bond breakage to produce intermediate M6 (*m*/*z* = 216). Through closed-loop reaction and hydroxylation, M6 was decomposed to intermediates M7 (*m*/*z* = 135) or M8 (*m*/*z* = 153) and then became small molecule products by a series of redox reactions. Similarly, according to the mass spectral data of TC-HCl at 40 min of visible light treatment (Figure S3b), the possible degradation path of TC-HCl in the photocatalytic system was reasonably deduced (Figure 10b) [1,7]. By losing N-methyl, TC-HCl molecules (*m*/*z* = 445) were converted to T1 (*m*/*z* = 431). With the deletion of -CH_3_ and O=C-NH_2_ of T1, T2 (*m*/*z* = 388) was formed. Then, T2 was decomposed into T3 (*m*/*z* = 362), T4 (*m*/*z* = 318), T5 (*m*/*z* = 249), and T6 (*m*/*z* = 170) following a series of ring opening, deacylation, deamination, and dehydroxylation reactions. Additionally, small molecule products T7 (*m*/*z* = 99), T8 (*m*/*z* = 102), and T9 (*m*/*z* = 131) were further generated. Finally, these small molecule compounds were mineralized to CO_2_ and H_2_O by the action of ROS.

#### 3.4.2. Roles of the Active Species

**Figure 11:**
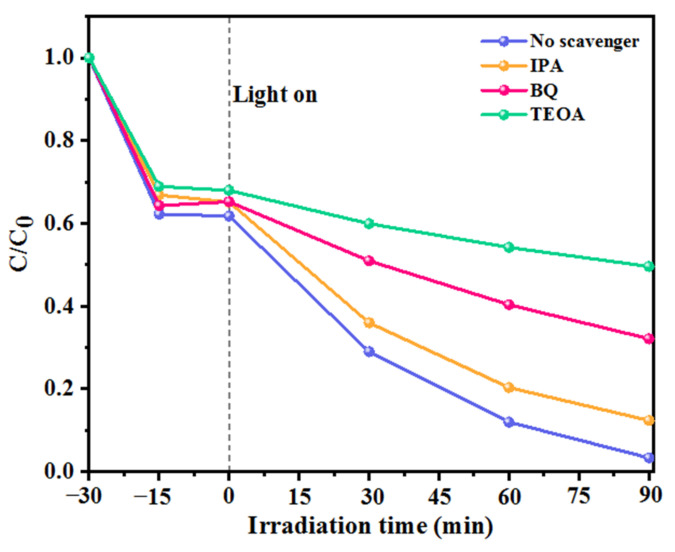
Effects of radical scavengers on the degradation of MB for CNAC-10 system.

Free radical capture experiments were performed to investigate the important role of ROS in the photocatalytic degradation of MB by CNAC-10. Isopropyl alcohol (IPA, 1 mM), p-benzoquinone (BQ, 1 mM), and triethanolamine (TEOA, 1 mM) were selected as ·OH, ·O_2_^−^, and h^+^ burst agents, respectively. As shown in Figure 11, the addition of IPA resulted in a slight but not significant decrease in MB removal from 96.5% to 87.6%, indicating that ·OH is not the major radical for the degradation of MB. However, the degradation efficiency of MB was remarkably reduced with the addition of BQ and TEOA, from 96.5% to 67.8% and 50.3%, respectively, indicating that ·O_2_^−^ and h^+^ play key roles in the MB photodegradation. According to the results of free radical capture experiments, it can be concluded that ·OH, ·O_2_^−^, and h^+^ all contribute to the removal of MB to varying degrees, and the order of the effect of ROS on MB photodegradation is h^+^ > ·O_2_^−^ > ·OH.

#### 3.4.3. Potential Photocatalytic Mechanism

**Figure 12:**
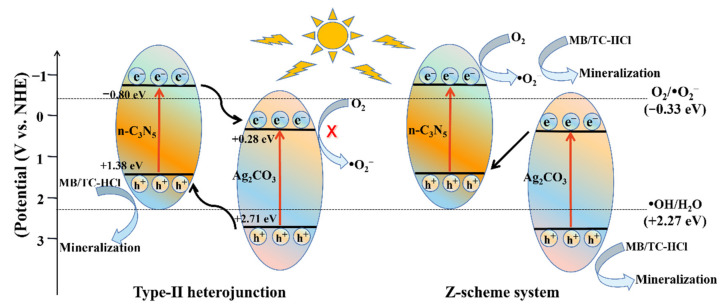
The proposed photocatalytic mechanism of MB and TC-HCl degradation by n-C_3_N_5_/Ag_2_CO_3_ composite.

It is critical to measure the band edge positions of semiconductors for researching the possible photocatalytic mechanism. Therefore, the energy band structures of n-C_3_N_5_ and Ag_2_CO_3_ were explored by XPS valence band spectroscopy. As shown in Figure S4a,b, the valence band (VB) positions of n-C_3_N_5_ and Ag_2_CO_3_ are located at about 1.38 and 2.71 eV, respectively. Then, the conduction bands (CB) of n-C_3_N_5_ and Ag_2_CO_3_ are −0.80 eV and 0.28 eV according to UV-vis DRS analysis with the equation *Eg* = E_VB_ − E_CB_. Two possible photogenerated carrier transfer mechanisms in the CNAC-10 system are revealed in Figure 12, namely type-II heterojunction and Z-scheme heterojunction. Based on the assumption that the photogenerated charge transfer mechanism between n-C_3_N_5_ and Ag_2_CO_3_ is consistent with the type-II heterojunction when the CNAC-10 catalyst is excited by visible light, the photogenerated electrons will be transferred from the CB of n-C_3_N_5_ to the CB of Ag_2_CO_3_ due to the higher CB and VB energy levels of n-C_3_N_5_ than the corresponding energy levels of Ag_2_CO_3_. Simultaneously, the photogenerated holes of Ag_2_CO_3_ will be transferred to the VB of n-C_3_N_5_. Although the formation of type-II heterojunction can improve the electron-hole separation efficiency of the photocatalyst, it is achieved at the cost of reducing its redox ability [32,68]. Since the reduction potential of Ag_2_CO_3_ is weaker than the standard redox potential of O_2_/·O_2_^−^ (−0.33 eV vs. NHE), the ·O_2_^−^ radical cannot be generated by the electrons on its CB [23]. Similarly, the photogenerated holes on the VB of n-C_3_N_5_ cannot react with H_2_O/OH^−^ to generate ·OH radical because the oxidation potential of n-C_3_N_5_ is lower than the standard redox potential of H_2_O/·OH (2.27 eV vs. NHE) [30]. It is obvious that the type-II heterojunction is contradicted by the results of radical trapping experiments. Therefore, the Z-scheme heterojunction transfer system was proposed. In the Z-scheme system, the electrostatic attraction between the electrons of Ag_2_CO_3_ and the holes of n-C_3_N_5_ makes the migration of photogenerated carriers physically more reasonable than that of type-II heterojunctions. When exposed to visible light, electrons generated on the CB of Ag_2_CO_3_ combine with holes on the VB of n-C_3_N_5_ to form a Z-scheme structure. In this way, the purpose of separating the electron-hole pairs is achieved, and the electrons and holes in the CNAC-10 system are concentrated on CB with a higher reduction potential and VB with a higher oxidation potential, respectively. The ·O_2_^−^ radical can be generated by the reaction of photogenerated electrons on n-C_3_N_5_ with oxygen molecules, while holes on Ag_2_CO_3_ can oxidize MB and TC-HCl directly or indirect by reacting with H_2_O/OH^−^ to form ·OH. The formation of Z-scheme heterojunction can be further verified by ESR analysis. From Figure S5a,b, it can be found that no signals of ·O_2_^−^ and ·OH are detected under dark conditions, while in the case of visible light irradiation, the ESR signal peaks of ·O_2_^−^ and ·OH can be clearly detected. On the whole, it is more plausible that the Z-scheme photogenerated charge transfer system is used to explain the action mechanism of ROS for CNAC-10 catalysts. According to the above analysis, the reaction Formula (3)–(6), which may occur in the system, is proposed:

CNAC-10 + hv → CNAC-10 (e^−^, h^+^) (3)

e^−^ (n-C_3_N_5_) + O_2_ → ·O_2_^−^ (4)

h^+^ (Ag_2_CO_3_) + H_2_O/OH^−^ → ·OH + H^+^ (5)

·O_2_^−^/·OH/h^+^ + MB/TC-HCl → CO_2_ +H_2_O. (6)

## 4. Conclusions

In this study, novel n-C_3_N_5_/Ag_2_CO_3_ composite photocatalysts were successfully prepared by thermal oxidative stripping and in situ deposition for the efficient degradation of MB and TC-HCl under visible light. According to the results of the characterization analysis and experiments, it can be assumed that a close interaction between n-C_3_N_5_ and Ag_2_CO_3_ occurred. Among the series of CNAC-X samples synthesized, CNAC-10 exhibited excellent recyclability and the highest photodegradation efficiency for MB and TC-HCl. Furthermore, the intermediates and possible degradation pathways of MB with TC-HCl were analyzed by LC-MS. The high photocatalytic activity of CNAC-10 was attributed to the synergistic effects of the ultrathin structure of the nanosheets, carbon defects, and Z-scheme heterojunctions. In conclusion, n-C_3_N_5_/Ag_2_CO_3_ nanocomposite is a novel photocatalyst that can effectively treat organic wastewater. Our work provides a new perspective for the design of novel and efficient C_3_N_5_-based photocatalysts and contributes to the development of low-cost and efficient photocatalysts for the control and remediation of environmental pollutants in the future.

## Data Availability

Data are contained within the article.

## Supplementary Materials

The following supporting information can be downloaded at: https://www.mdpi.com/article/10.3390/nano12152701/s1, Figure S1: The N_2_ adsorption-desorption isotherms of g-C_3_N_5_ and n-C_3_N_5_; Figure S2: The XPS measurement spectra of the CNAC-10 composite; Figure S3: (a) *m*/*z* values of MB photodegradation at 90 min in the presence of CNAC-10 catalyst, (b) *m*/*z* values of TC-HCl photodegradation at 40 min in the presence of CNAC-10 catalyst; Figure S4: (a,b) The VB-XPS spectra of n-C_3_N_5_ and Ag_2_CO_3_, respectively; Figure S5: ESR signals of (a) ·O_2_^−^ and (b) ·OH generated by CNAC-10; Table S1: Comparation of the photocatalytic performance of photocatalyst composites.
