# Supplementary material for: In Situ Coupling Carbon Defective C3N5 Nanosheet with Ag2CO3 for Effective Degradation of Methylene Blue and Tetracycline Hydrochloride
Source: Nanomaterials (Basel). 2022 Aug 5;12(15):2701. doi: 10.3390/nano12152701 (PMC9370685; doi:10.3390/nano12152701)
Supplement: Supplementary file 1 [file nanomaterials-12-02701-s001.zip › nanomaterials-1835993-supplementary.pdf]

nanomaterials

MDPI

Supporting Information

In Situ Coupling Carbon Defective C_3N_5 Nanosheet with Ag_2CO_3 for Effective Degradation of Methylene Blue and Tetracycline Hydrochloride

Guoyu Li [1], Genying Zeng [1], Zhangkai Chen [1], Jiaming Hong [1], Xiaodong Ji [1], Zhiqiang Lan [1], Xiaofei Tan [2,3], Meifang Li [1], Xinjiang Hu [1,*] and Chunfang Tang [1,*]

[1] College of Environmental Science and Engineering, Central South University of Forestry and Technology, Changsha 410004, China
[2] College of Environmental Science and Engineering, Hunan University, Changsha 410082, China
[3] Key Laboratory of Environmental Biology and Pollution Control, Hunan University, Ministry of Education, Changsha 410082, China
* Correspondence: xjhu@csuft.edu.cn (X.H.); cshntcf@163.com (C.T.)

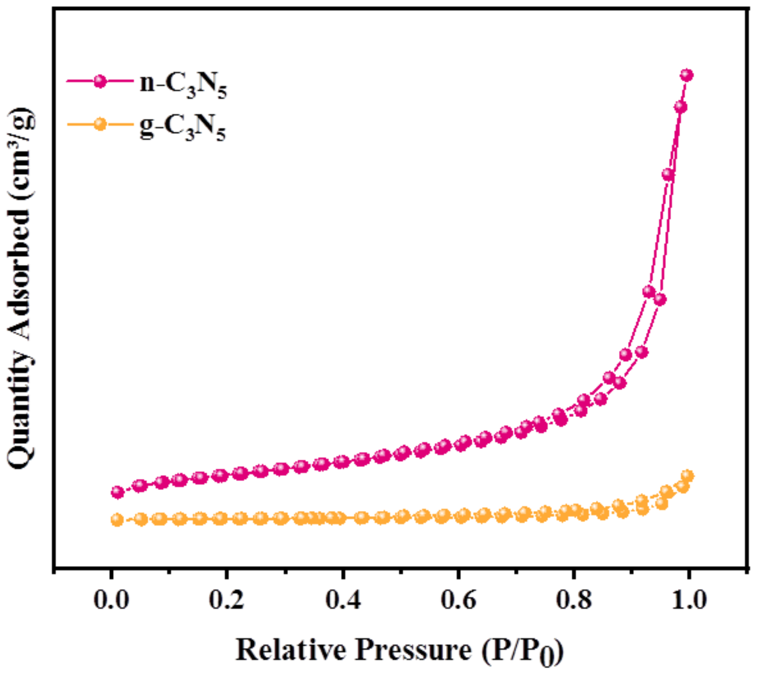

Figure S1. The N_2 adsorption-desorption isotherms of g-C_3N_5 and n-C_3N_5.

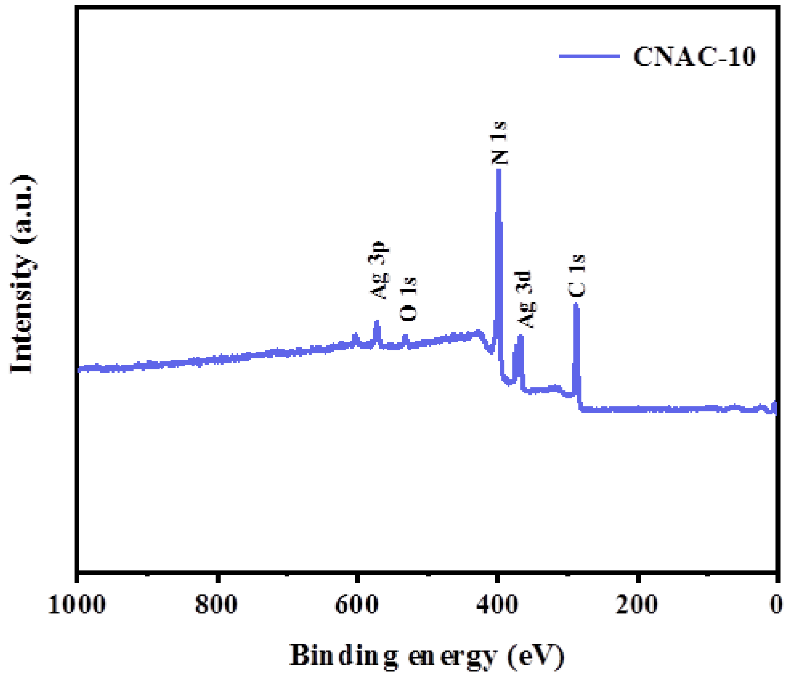

Figure S2. the XPS measurement spectra of the CNAC-10 composite.

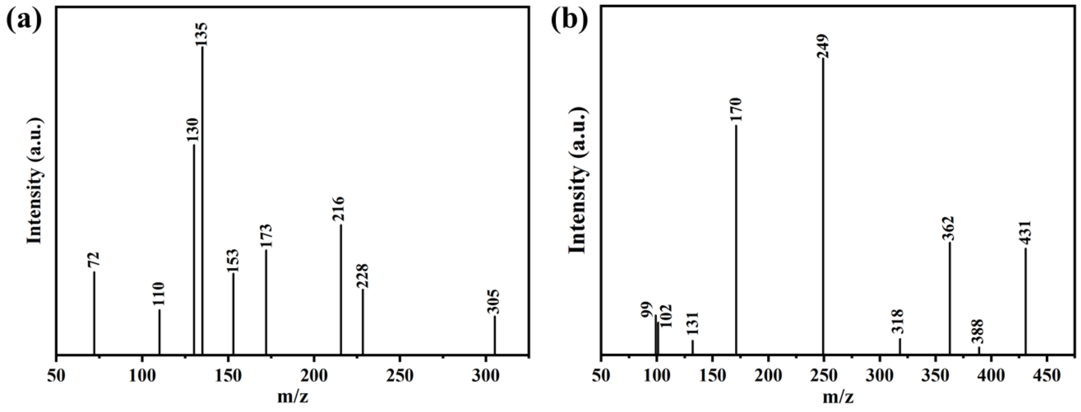

Figure S3. (**a**) m/z values of MB photodegradation at 90 min in the presence of CNAC-10 catalyst, (**b**) m/z values of TC-HCl photodegradation at 40 min in the presence of CNAC-10 catalyst.

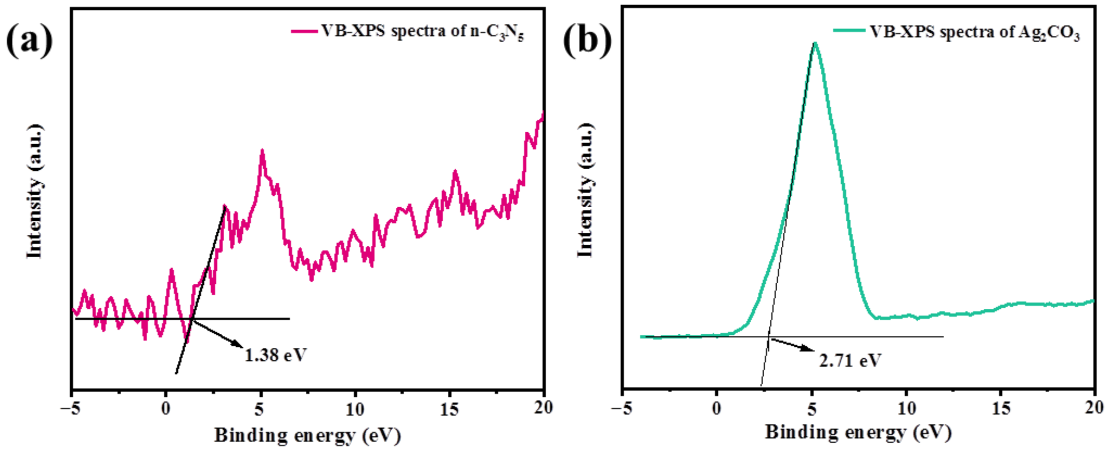

Figure S4. (**a**,**b**) The VB-XPS spectra of n-C_3N_5 and Ag_2CO_3, respectively.

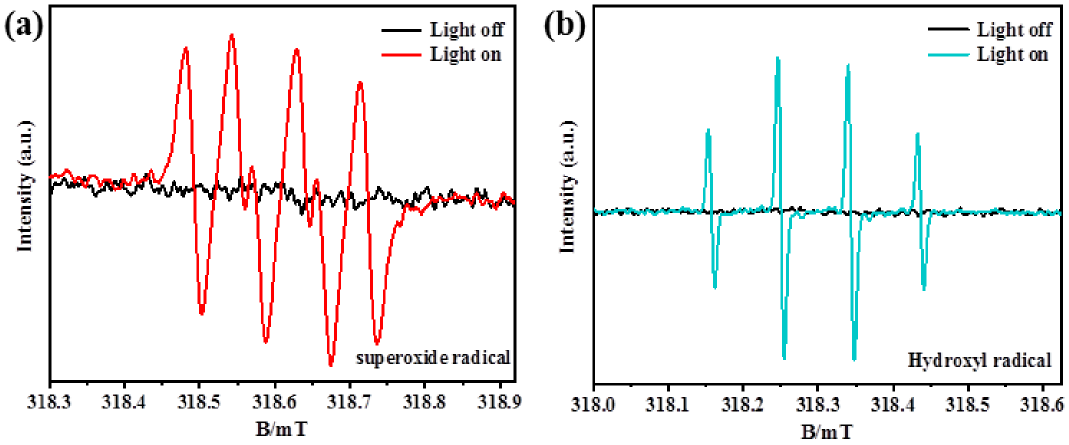

Figure S5. ESR signals of (**a**) •O_2^- and (**b**) •OH generated by CNAC-10.

Table S1. Comparation of the photocatalytic performance of photocatalyst composites.

photocatalyst	Contaminants	Photodegradation rate	Concentration of photocatalyst (g·L^{-1})	Ref.
**n-C_3N_5/Ag_2CO_3**	MB=60 mg/L	96.5% in 90 min	1	This work
**n-C_3N_5/Ag_2CO_3**	TC-HCl=50 mg/L	97.6% in 100 min	0.4	This work
**Bi_2WO_6@C_3N_5**	TC=20 mg/L	92.7% in 60 min	0.2	[1]
**AgCl/g-C_3N_5**	RhB=10 mg/L	85% in 60 min	1	[2]
**Ag_3PO_4@g-C_3N_5**	TC-HCl=20 mg/L	90.5% in 60 min	1	[3]
**Bi_2WO_6@g-C_3N_5**	TC=10 mg/L	99.8% in 60 min	0.4	[4]
**Ag_2CO_3@g-C_3N_4**	MB=10 mg/L	95.0% in 54 min	1	[5]

References:

[1] Li, S., Cai, M., Liu, Y., Zhang, J., Wang, C., Zang, S., Li, Y., Zhang, P. and Li, X. In situ construction of a C_3N_5 nanosheet/Bi_2WO_6 nanodot S-scheme heterojunction with enhanced structural defects for the efficient photocatalytic removal of tetracycline and Cr(vi). *Inorg. Chem. Front*. **2022**, 911, 2479-2497.

[2] Vadivel, S., Hariganesh, S., Paul, B., Rajendran, S., Habibi-Yangjeh, A., Maruthamani, D. and Kumaravel, M. Synthesis of novel AgCl loaded g-C_3N_5 with ultrahigh activity as visible light photocatalyst for pollutants degradation. *Chem. Phys. Lett*. **2020**, 738, 136862.

[3] Yin, H., Cao, Y., Fan, T., Zhang, M., Yao, J., Li, P., Chen, S. and Liu, X. In situ synthesis of Ag_3PO_4/C_3N_5 Z-scheme heterojunctions with enhanced visible-light-responsive photocatalytic performance for antibiotics removal. *Sci. Total. Environ*. **2021**, 754, 141926.

[4] Saravanakumar, K., Maheskumar, V., Yea, Y., Yoon, Y., Muthuraj, V. and Park, C. M. 2D/2D nitrogen-rich graphitic carbon nitride coupled Bi_2WO_6 S-scheme heterojunction for boosting photodegradation of tetracycline: Influencing factors, intermediates, and insights into the mechanism. *Compos. Part. B Eng*. **2022**, 234, 109726.

[5] An, W., Sun, K., Hu, J., Cui, W. and Liu, L. The Z-scheme Ag_2CO_3@g-C_3N_4 core-shell structure for increased photoinduced charge separation and stable photocatalytic degradation. *Appl. Surf. Sci*. **2020**, 504, 144345.
